# NSUN7 Regulates Sperm Flagella Formation at All Stages of Spermiogenesis

**DOI:** 10.3390/ijms27010257

**Published:** 2025-12-25

**Authors:** Vitaly S. Buev, Ekaterina A. Guseva, Maria P. Rubtsova, Anastasia V. Priymak, Svetlana E. Novikova, Olga A. Averina, Oleg A. Permyakov, Olga O. Grigoryeva, Vasily N. Manskikh, Victor G. Zgoda, Olga A. Dontsova, Petr V. Sergiev

**Affiliations:** 1 Faculty of Chemistry, Lomonosov Moscow State University, 119991 Moscow, Russiamprubtsova@gmail.com (M.P.R.);; 2 Faculty of Bioengineering and Bioinformatics, Lomonosov Moscow State University, 119991 Moscow, Russia; 3 Belozersky Institute of Physico-Chemical Biology, Lomonosov Moscow State University, 119991 Moscow, Russia; 4 Institute of Biomedical Chemistry, 119121 Moscow, Russiavictor.zgoda@gmail.com (V.G.Z.); 5 Shemyakin–Ovchinnikov Institute of Bioorganic Chemistry, 117997 Moscow, Russia

**Keywords:** NSUN7, spermatozoa, infertility, molecular reproduction, flagella assembly, longitudinal columns, spermatid sorting

## Abstract

Spermiogenesis requires extensive molecular and structural remodeling to produce motile sperm. Mutations in the testis-specific RNA methyltransferase NSUN7 are associated with defective fibrous sheath, impaired sperm motility, and male infertility. However, the underlying molecular mechanisms remain poorly understood. Here, we performed proteomic profiling of sorted, elongated, and round spermatids, as well as mature spermatozoa from Nsun7 knockout mice. We showed that NSUN7 is present at all stages of spermiogenesis and is most abundant in round spermatids, which corresponds to the formation of the flagellum and fibrous sheath assembly. Loss of NSUN7 altered the abundance of proteins essential for dynein arm assembly (PIH1D3, CCDC103, CCDC40), intraflagellar transport (IFT122), and fibrous sheath organization (AKAP3, AKAP4, ROPN1L). We also showed that the previously detected impaired retention of cytoplasm in elongated spermatids may be caused by plectin accumulation. Interestingly, no statistically significant changes were found in mature sperm proteomes upon Nsun7 inactivation. Our findings support a model in which NSUN7 primarily stabilizes protein complexes and coordinates flagellar assembly. This indicates that NSUN7 is a critical regulator of spermiogenesis, and its malfunction is a contributing factor to male infertility.

## 1. Introduction

Spermatogenesis is a complex process by which male germ cells (spermatocytes) differentiate into spermatozoa. This process involves mitotic divisions and significant cell rearrangements [1,2]. The post-meiotic stages of spermatogenesis, including the differentiation of round spermatids into elongated spermatids and subsequently into mature spermatozoa, are collectively referred to as ‘spermiogenesis’. During this process, the cells undergo extensive biochemical and morphological changes, including chromatin condensation [3], conversion of the Golgi apparatus into an acrosome [2,4], flagellum assembly, and the loss of 90% of the cytoplasm [5].

These morphological rearrangements are accompanied by significant changes in gene expression [6] and cellular protein composition [7], enabling a cell to modify its molecular composition for subsequent transformations. Specifically, the round spermatid stage is marked by acrosome formation [8] and is characterized by proteins essential for this process, such as ACRV1 (Acrosomal Vesicle Protein 1) [9]. Additionally, at the round spermatids stage, structural elements surrounding the axoneme, such as circumferential ribs and longitudinal columns (LC) of fibrous sheath, start to assemble [10]. Subsequently, during the elongated spermatid stage, the annulus, a ring of septin molecules surrounding the base of the axoneme, forms, while fully formed circumferential ribs and LC attach to the axoneme and each other [10,11,12]. Consequently, this later stage involves proteins critical for these processes, including SPTX1 (Spermatid-Specific Thioredoxin-1 Protein) [13] and CBY3 (Chibby Family Member 3) [14].

One of the central processes of sperm maturation is DNA hypercondensation, which begins at the round spermatid stage and is completed by the elongated spermatid stage. This results in the formation of a transcriptionally inactive nucleus in the elongated spermatid [15], meaning translation occurs on the accumulated RNA synthesized earlier [15]. However, during the transition from round to elongated spermatids, nearly 14% of proteins change, altering more than 250 different pathways [7]. Accordingly, spermatids must have a mechanism for temporarily silencing RNA that will be translated at later stages. This is achieved through post-translational modifications and RNA-binding proteins. RNA modifications regulate the fate of RNA by affecting its splicing, translation level, and overall stability [16,17,18]. The NSUN (NOP2/Sun RNA methyltransferase) family of enzymes catalyzes m^5^C modifications in various RNAs, and several members of this family have been associated with germ cell development and fertility [19,20]. Another NSUN methyltransferase, NSUN7 (NOP2/Sun RNA methyltransferase family member 7), catalyzes m^5^C modifications [21] and downregulates a set of mRNAs associated with sperm flagellum motility at the elongated spermatid stage, thereby apparently suppressing the synthesis of these proteins in mature sperm [22].

Mutations in NSUN7 have been observed to contribute to male infertility in humans [19,23] and mice [22]. At the phenotypic level, the most notable abnormality caused by NSUN7 mutations is an alteration to the pattern of attachment of the fibrous sheath columns to the axoneme, which disrupts flagellar function and leads to impaired sperm motility [22]. Interestingly, this phenotype is only caused by a complete loss of the protein; meanwhile, mutant mice carrying the presumably catalytically inactive variant demonstrate a normal fertility rate [24]. This striking finding suggests that NSUN7’s catalytic activity might be dispensable for fertility, implicating its non-enzymatic, structural, or protein interaction hub functions in proper fibrous sheath assembly. Despite this evidence, the proteomic landscape of maturing spermatids in relation to NSUN7 deficiency, which could elucidate the specific pathways and protein interactions dependent on NSUN7, remains unstudied.

To investigate the hypothesis that NSUN7 regulates spermiogenesis and flagellar assembling on the protein level, we performed a comparative proteomic analysis of spermatozoa, round spermatids, and elongated spermatids isolated from *Nsun7*-knockout and wild-type mice. We showed that NSUN7 is tightly associated with dynein arm assembly and intraflagellar transport (IFT) mechanisms. Disruption to these processes caused by *Nsun7* inactivation leads to male subfertility and mispositioning of the LC of the fibrous sheath.

## 2. Results

### 2.1. NSUN7 Is Present in Developing Spermatids and Mature Sperm

**Figure 1:**
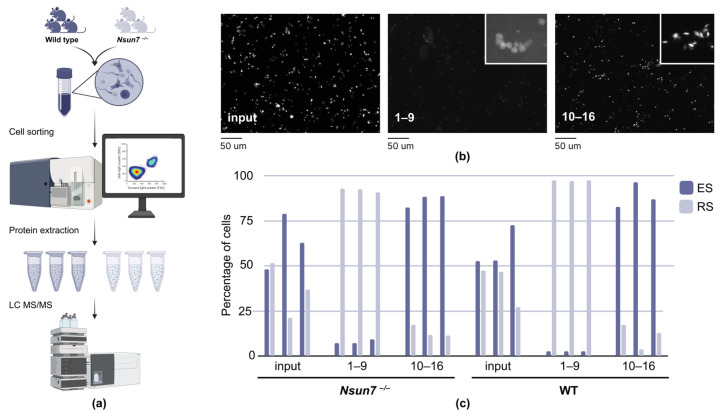
Sorting of round and elongated spermatids. (**a**) General scheme of cell enrichment and following sample preparation for tandem mass spectrometry. (**b**) Micrographs of testicular cells before sorting (input) and of obtained enriched fractions (numbers indicate stages of spermiogenesis; 1–9—round spermatids, 10–16—elongated). (**c**) Bar plot illustrating proportion of cell types in samples prior to (input) and subsequent to (1–9 and 10–16 fractions) cell sorting of wild-type (*n* = 3, at age of 2 months) and *Nsun7^−/−^* (*n* = 3, at age of 2 months) testicular cells.

In order to study in detail how NSUN7 affects various molecular mechanisms necessary for the proper development of sperm, we aimed to analyze the protein composition of sperm precursors. To this end, we applied the method of testicular cell sorting [25] (Figure 1a). The spermatid separation method is based on apparent DNA content (SYTO16 staining), cell size, and granulosity. SYTO16 provides high-efficiency staining for both decondensed and condensed DNA, a critical feature when analyzing spermatogenic cells. Additionally, SYTO16 fluorescence remains stable at 4 °C, allowing for extended cell sorting without diminishing signal intensity [25].

This method enables the purification of four distinct populations of spermatogenic cells, corresponding to stages 1–9, 10–12, 13–14, and 15–16 of mouse spermatogenesis [26]. As mutations in *Nsun7* are associated with abnormalities in sperm motility [22,27] and LC mispositioning, in our study, we concentrated on its effect on round (RS, 1–9 stages of spermatogenesis) and elongated spermatids (ES, 10–16 stages of spermatogenesis). The reason for this is that, in round spermatids, LCs start to assemble, while in elongated spermatids, they are being attached to the axoneme and circumferential ribs.

In order to control the purity of the enriched fractions obtained, microscopy was performed on each sample before (input) and after sorting. The application of this technique resulted in the isolation of fractions enriched in a specific cell type, with a purity level exceeding 75% (Figure 1b,c).

**Figure 2:**
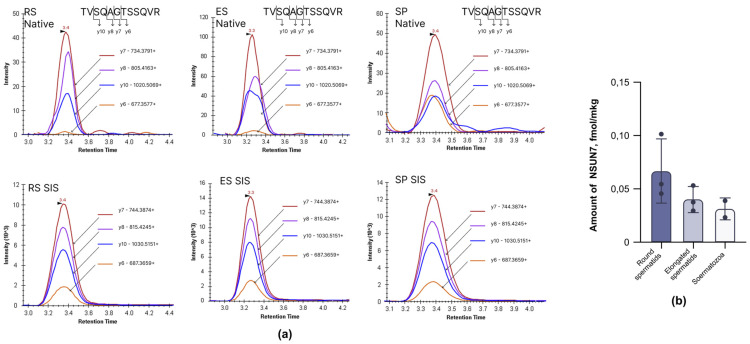
Levels of Nsun7 in cells during the late stages of spermatogenesis. (**a**) Trace of SRM transitions for native TVSQAGTSSQVR peptide (upper panel) and its stable isotope-labeled peptide standards (SIS) (lower panel) detected in round spermatids (RS), elongated spermatids (ES), and spermatozoa (SP) samples. The *x*-axis shows the retention time. The *y*-axis shows fragment ion intensity. The figure shows a match in retention time and fragmentation pattern for the SIS and its natural counterpart. (**b**) Bar plot representing concentrations of NSUN7 after trypsin digestion of total peptides extracted from round spermatids (RS), elongated spermatids (ES), and spermatozoa (SP) of wild-type mice (*n* = 3).

To obtain a sufficient amount of material for further analysis, we pooled the samples from three mice. Following the sorting and lysis of the obtained cells, the amount of NSUN7 in each fraction was estimated using targeted mass spectrometry analysis of NSUN7 with a stable-isotope-labeled (13C,15N-Arg) standard (SIS) peptide, TVSQAGTSSQVR, which had previously been shown to be specific for NSUN7 detection [22] (Figure 2a). NSUN7 was detected in the extracts of cells at all stages of late spermatogenesis (Figure 2b). During the round spermatid stage, with the onset of LC formation, the level of NSUN7 reached its maximum, corresponding to 0.0637 ± 0.03 fmole/μg total peptides after trypsin digestion. As spermatids matured, there was a gradual decline in NSUN7 concentration, with a minimum observed in mature sperm (0.0295 ± 0.01 fmole/μg total peptides). Thus, NSUN7 could regulate LC formation at all stages.

### 2.2. NSUN7 Influence on Transcription and Axoneme Associated Proteins in Round Spermatids

Previous studies have shown that NSUN7 affects the proteome of the whole testis [22]. However, due to the limitations of the LC-MS/MS method and heterogeneity of proteins in different cell types, some significant stage-specific differences may have been overlooked. In this study, we investigated the influence of NSUN7 on the proteome of cells at different stages of spermatogenesis using the above-described technique. In order to obtain enough cells for analysis, we pooled testes from 3 mice in each sample.

**Figure 3:**
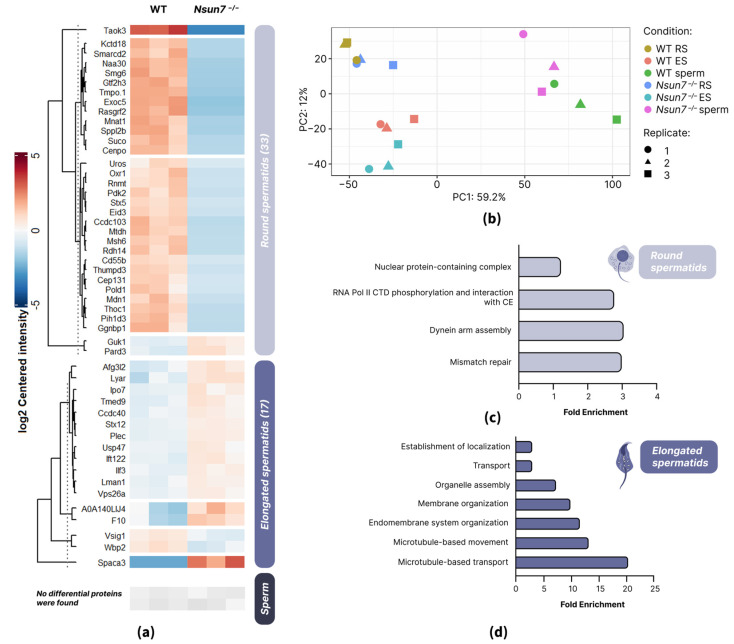
NSUN7 regulates proteomic changes during spermiogenesis. (**a**) Heatmap showing proteins whose abundance is significantly different in mature sperm, round spermatids, and elongated spermatids of the wild-type (WT) (*n* = 3, at the age of 2 months) and *Nsun7^−/−^* (*n* = 3, at the age of 2 months) mice. (**b**) PCA plot of LC-MS/MS data from spermatozoa (sperm), round spermatids (RS), and elongated (ES) spermatids of wild type (WT) (*n* = 3, at the age of 2 months) and *Nsun7^−/−^* (*n* = 3, at the age of 2 months) mice. (**c**) GO term enrichment analysis of DEP of round spermatids. (**d**) GO term enrichment analysis of DEP of elongated spermatids.

Panoramic LC-MS/MS analysis of the proteomes of spermatozoa, round spermatids, and elongated spermatids revealed distinct, stage-specific protein profiles. Despite some minor cellular heterogeneity within the sorted fractions, as indicated by microscopy (Figure 1b,c), principal component analysis (PCA) of the proteomic data revealed a clear separation of the samples by cell type (Figure 3b).

In a fraction of round spermatids, we identified 2654 proteins in total. Overall, we revealed 33 differentially expressed proteins (DEPs), 2 of which were upregulated and 31 of which were downregulated in *Nsun7^−/−^* mice (Figure 3a and Figure S1a). Among DEPs, we observed proteins involved in the initiation of the export of polyadenylated RNA (THO complex subunit 1—THOC1) and the phosphorylation of RNA polymerase II C-terminal domain (CTD) (MNAT1 component of CDK activating kinase—MNAT1, RNA guanine-7 methyltransferase—RNMT and General transcription factor IIH subunit 3—GTF2H3), as well as proteins associated with mismatch repair (DNA polymerase delta 1—POLD1 and mutS homolog 6—MSH6) (Figure 3c and Figure S1a). 

**Figure 4:**
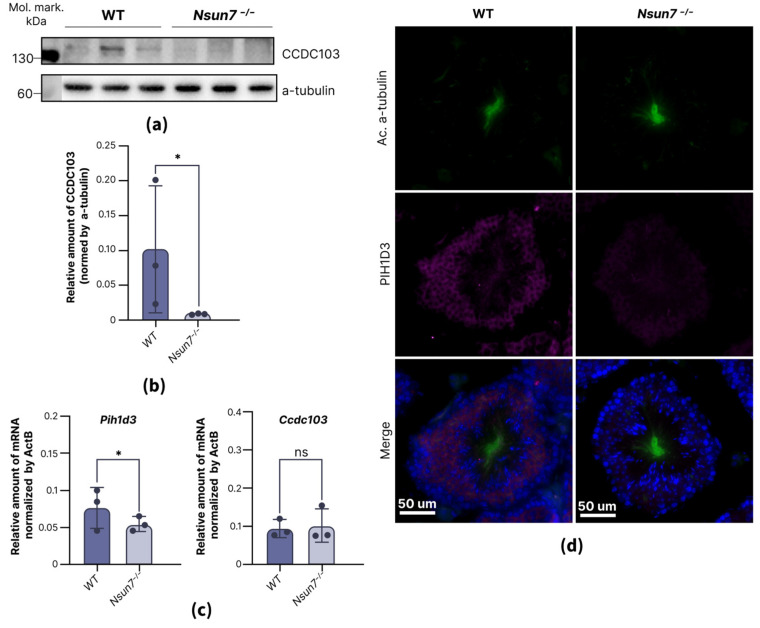
NSUN7 stabilizes CCDC103 and PIH1D3 in round spermatids. (**a**) Comparative immunoblotting of round spermatid lysates of wild-type (left 3 lanes) and *Nsun7^−/−^* (right 3 lanes) mice. Antibodies against CCDC103 and control antibodies against α-tubulin were used, as indicated to the right of the panels. (**b**) Bars showing α-tubulin normalized level CCDC103 in wild-type and *Nsun7^−/−^* mice according to immunoblotting (**a**). Means ± standard deviation are shown (*n* = 3). Results of the unpaired *t*-test are shown, *—*p*-value < 0.05; ns—not significant. (**c**) Comparison of the mRNA levels of Pih1d3 and Ccdc103 in wild-type (WT, *n* = 3) and *Nsun7^−/−^* (*n* = 3) mice. (**d**) Immunostaining with anti-PIH1D3 and anti-acetylated-α-tubulin (Ac. α-tubulin) antibodies of testis cross-sections from wild-type and *Nsun7^−/−^* mice.

The most striking finding, however, is the downregulation of proteins involved in the assembly of the dynein arms (PIH1 domain-containing protein 3—PIH1D3 and coiled-coil domain containing 103 protein—CCDC103) in *Nsun7^−/−^* mice (Figure 4a–c and Figure S5). It has previously been demonstrated that disturbances in these proteins lead to reduced flagellar motility, primarily due to the disorganization of dynein motor assembly, but abnormalities in the composition of the fibrous sheath can also be seen [28,29]. Intriguingly, certain *CCDC103* missense mutations in humans cause fibrous sheath columns to misposition, attaching to doublets 2–8 instead of 3–8 [28], a phenotype that mirrors the anomalies observed in *Nsun7^−/−^* mice [22]. 

According to the studied clinical case, pathogenic mutations in the *CCDC103* gene lead to a diminished CCDC103 protein level [28]. We confirmed the decrease in CCDC103 abundance at the protein level, but not at the RNA level, in round spermatids of *Nsun7^−/−^* mice using Western blotting (Figure 4a,b) and real-time PCR (Figure 4c). In later spermatogenic stages, the protein level of CCDC103 fell below detectable levels, preventing a reliable evaluation of NSUN7’s effect on its expression (Figures S1b,c and S7).

The inactivation of the cytoplasmic dynein axonemal assembly factor *Pih1d3* has been shown to result in immotile spermatozoa with morphological abnormalities, such as folded tails [29]. The same morphological changes were reported for *Nsun7^−/−^* [22]. We observed *Pih1d3* expression in the cytoplasm of spermatocytes and round spermatids, where its level was lower in *Nsun7^−/−^* than in wild-type mice (Figure 4d). Similar to that for Ccdc103 mRNA, the Pih1d3 mRNA level was not altered (Figure 4c).

Furthermore, as NSUN7 has been shown to be an RNA-binding protein, we compared the observed proteomic changes in round spermatids with the RNA cross-linking efficiency profile of NSUN7 [22] (Figure S1d). Given that no correlation was observed, and that the RNA levels of Ccdc103 and Pih1d3 in round spermatids remained unaltered, NSUN7 may instead regulate the translation of its protein effectors or be involved in maintaining their stability.

### 2.3. NSUN7 Influences Transport Proteins in Elongated Spermatids, While Proteome of Mature Sperm Remains Unaltered

When analyzing samples of elongated spermatids, we detected 1380 proteins in total. Of these, 17 were DEPs, two of which were downregulated and 15 of which were upregulated in *Nsun7^−/−^* mice (Figure 3a). 

Interestingly, six of the identified DEPs are associated with membrane organization (syntaxin-12—STX12, sperm acrosome membrane-associated protein 3—SPACA3, transmembrane p24 trafficking protein 9—TMED9, platelet-activating factor acetylhydrolase IB subunit alpha—PAFAH1B1, AFG3 ATPase family gene 3-like 2—AFG3L2, and plectin—PLEC), while ten are associated with intracellular transport and cell motility (lectin mannose-binding 1—LMAN1, PAFAH1B1, STX12, coiled-coil domain-containing protein 40—CCDC40, intraflagellar transport 122—IFT122, TMED9, importin-7—IPO7, Ly1 antibody reactive—LYAR, AFG3L2, and SPACA3) (Figure 3d and Figure S2a,b). Furthermore, four hits (IFT122, heavy chain 14 of axonemal dynein—DNAH14 or A0A140LIJ4, PAFAH1B1, and CCDC40) (Figure 3d and Figure S2a) are involved in microtubule-based movement, showing a strong correlation with the observed phenotype of the knockout. 

**Figure 5:**
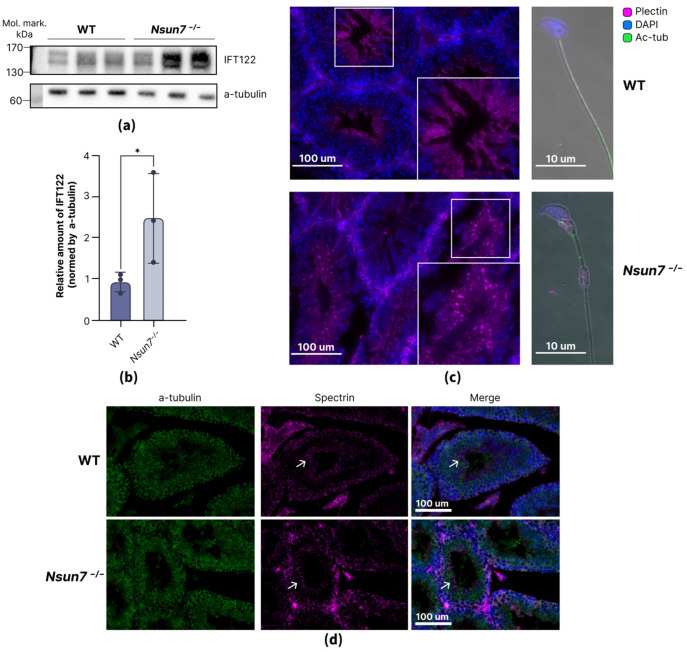
NSUN7 influence on the components of the intraflagellar transport and tubulobulbar complexes. (**a**) Comparative immunoblotting of elongated spermatid lysates of wild-type (left 3 lanes) and *Nsun7^−/−^* (right 3 lanes) mice. Antibodies against IFT122 and control antibodies against α-tubulin were used as indicated right to the panels. (**b**) Bars showing α-tubulin normalized levels of IFT122 in wild-type and *Nsun7^−/−^* mice according to immunoblotting (**a**). Means ± standard deviation are shown (*n* = 3). Results of the unpaired *t*-test are shown, *—*p*-value < 0.05. (**c**) Immunostaining with anti-plectin and anti-acetylated-α-tubulin antibodies of testis cross-sections (left panel) and extracted elongated spermatids (right panel) from wild-type and *Nsun7^−/−^* mice. Cross-sections also include a magnified image of a seminiferous tubule central part, where the plectin granules could be observed. (**d**) Immunostaining with anti-spectrin and anti-α-tubulin antibodies of testis cross-sections from wild-type and *Nsun7^−/−^* mice. Arrows indicate spectrin localization.

The last group of proteins is of the greatest interest, as it includes the structural chain of the dynein (DNAH14), and the protein that affects its motor function and activity (PAFAH1b1) [30], as well as the protein that is essential for assembling the dynein arms (CCDC40) [31]. IFT122 is a part of the IFT-A complex, which mediates both retrograde trafficking towards the base of flagella driven by dynein-2 [32,33] and the ciliary import of various membrane proteins across the ciliary gate [34]. We proved that NSUN7 affects the level of IFT122 in elongated spermatids using Western blotting (Figure 5a,b and Figure S6) and immunostaining (Figure S2c). Interestingly, at earlier stages of spermatogenesis, we observed no significant effect of NSUN7 on IFT122 levels (Figures S2d,e and S8).

Another striking observation is the upregulation of plectin in *Nsun7^−/−^* mice. Plectin is responsible for bridging microtubules with intermediate filaments and enhancing the formation of cytoskeletal systems in the cells [35]. In spermatogenic cells, plectin is involved in the stabilization of tubulobulbar complexes [36,37], which is needed for the elimination of residual cytoplasm [38]. We have demonstrated that plectin is expressed late in spermatogenesis and is localized in the spermatid body but not in the tail (Figure 5c). Microphotographs of testis cross-sections from wild-type and *Nsun7^−/−^* mice revealed a difference in plectin localization pattern. In the knockout mice, plectin accumulates in bright granules in the residual cytoplasm of forming spermatids, whereas in the wild type, it is diffusely distributed throughout the cell (Figure 5c). According to our previous studies, *Nsun7^−/−^* mice exhibit a higher percentage of spermatozoa that retain the residual body in a mature state [22]. Whether plectin accumulation causes the cytoplasm to be less easily detached or is itself the result of another disrupted mechanism remains unclear. To gain a better understanding, we performed an immunostaining analysis of testicular cross sections for spectrin (Figure 5d), which is also present in networks surrounding tubulobulbar complexes [37]. However, no significant changes in localization or protein levels were observed. This finding supports the hypothesis that the observed morphological abnormalities are predominantly the result of plectin accumulation.

We also compared proteomes of mature sperm of wild-type and *Nsun7^−/−^* mice. Although in total we identified 1718 proteins, none of them exhibited a significant difference in abundance between the studied mouse lines (Figure 3a and Figure S3a).

Finally, we compared the observed proteomic changes in elongated spermatids and spermatozoa with the efficiency of RNA cross-linking to the NSUN7 [22] (Figures S2f and S3b). As with round spermatids, no significant correlations were found.

### 2.4. NSUN7 May Regulate Protein Stabilization During Late Stages of Spermatogenesis

During spermatogenesis, the protein composition changes dramatically as the cell progresses through its different stages. Thus, we also analyzed how NSUN7 affects these changes in protein amounts. To identify trends, we traced differences in protein abundance between *Nsun7^−/−^* and wild-type mice from the round spermatid stage to mature spermatozoa. Only those trends where the fold-change between round spermatids and mature spermatozoa exceeded two-fold and showed a consistent direction (increase or decrease) across all stages were considered. In total, we identified 188 proteins exhibiting such fold-change trends (Figure S4a,b).

**Figure 6:**
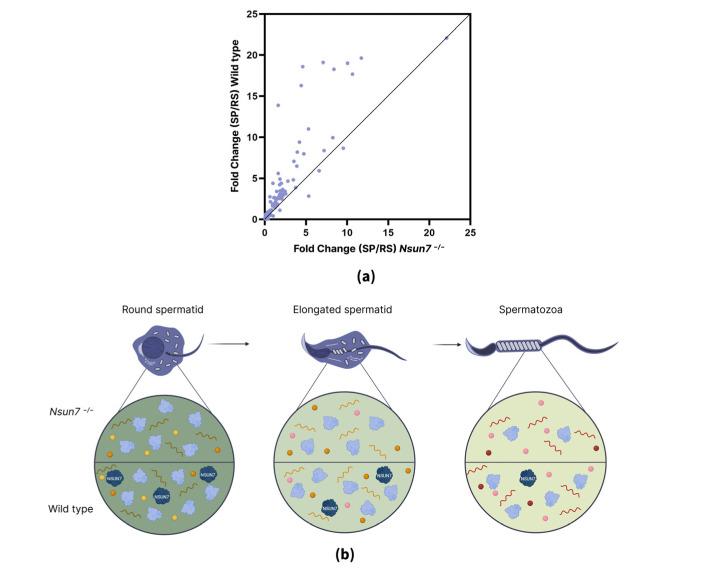

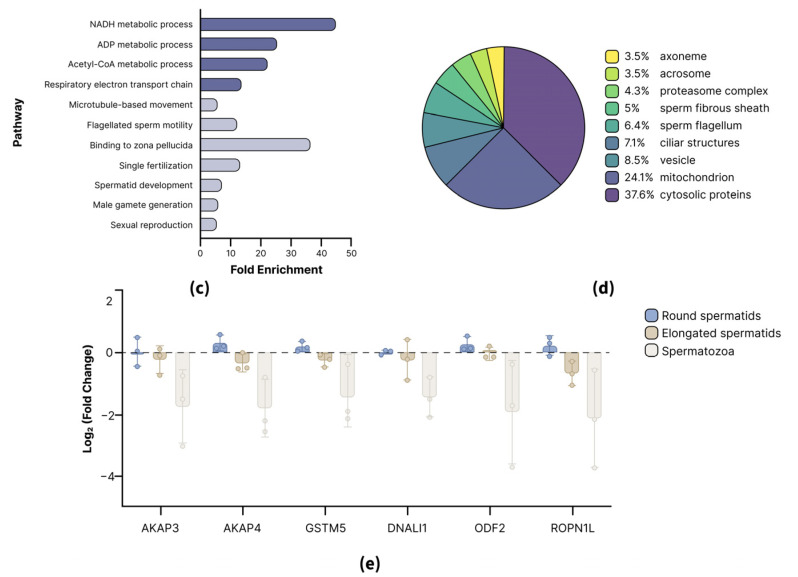
Trends of protein FCs affected by NSUN7. (**a**) Scatter plot of fold changes in protein amount between round spermatids and spermatozoa in wild-type and *Nsun7^−/−^* mice. (**b**) Possible scheme describing observed decreasing trends in protein FC. (**c**) GO term enrichment analysis of the proteins with decreasing trends in FCs. (**d**) Pie chart showing localization of proteins with decreased FC trends. (**e**) FC of flagella-associated proteins in spermatozoa, round spermatids, and elongated spermatids.

Interestingly, the fold change (FC) of the vast majority of proteins (141) decreased as spermatogenesis progressed. Moreover, the amount of 105 proteins was almost identical in round spermatids in both mouse lines (|FC| < 0.5, |FC|—module of Fold Change); however, the difference in their level between *Nsun7^−/−^* and wild-type mice gradually increased in elongated spermatids and spermatozoa. One possible explanation is that proteins degraded faster or accumulated more slowly during spermiogenesis in *Nsun7^−/−^* mice compared to wild-type mice (Figure 6a). This suggests that NSUN7 likely regulates protein or RNA stability during spermatogenesis (Figure 6b). To explore the potential biological impact of this dysregulation, we analyzed the functional annotations of the affected proteins. Notably, proteins with decreasing differences in protein abundance between *Nsun7^−/−^* and wild-type mice were enriched for specific cellular components: 3.55% localize to the acrosome, and 21.98% are associated with cilia (Figure 6d). The subset of affected proteins includes key structural proteins of the fibrous sheath, such as A-kinase anchor protein 4 (AKAP4) and A-kinase anchor protein 3 (AKAP3), as well as ropporin-1-like protein (ROPN1L), which is involved in PKA-dependent signaling associated with the fibrous membrane (Figure 6c–e). The obtained results were further supported by immunoblotting for AKAP4 across three developmental stages (Figures S4e–h and S9–S11). Dynein axonemal light intermediate chain 1 (DNALI1) is of particular interest; mice with mutations in this gene exhibit the same structural changes to the organization of the fibrous membrane as *Nsun7* knockouts [39] (Figure 6e).

Most of the proteins showing an upward trend are not directly related to spermatogenesis, but are instead associated with protein translation and transport (Figure S4c,d).

## 3. Discussion

The methyltransferase NSUN7 is primarily recognized for its role in cancer, where it methylates the mRNA of CCDC9B (Coiled-Coil Domain Containing 9B) and reduces MYC expression [40]. Beyond oncology, NSUN7 has been strongly associated with male infertility [22,41], although the precise mechanisms underlying its contribution remain poorly understood. Missense mutations in this gene are associated with reduced sperm motility, abnormal morphology (including midpiece bending and retention of residual cytoplasm), and defective flagellar ultrastructure [21].

Our study reveals that NSUN7 exerts a multifaceted role in spermiogenesis, impacting both the structural assembly of the sperm flagellum and the stability of proteins throughout late germ-cell differentiation. By applying cell-type-specific proteomic profiling, we demonstrate that the loss of NSUN7 consistently decreased the concentration of proteins linked to dynein arm assembly, IFT, and fibrous sheath organization.

Assembly of sperm motility structures begins in round spermatids, when the axoneme forms and the formation of the fibrous sheath LC is initiated [10]. At this same stage, NSUN7 expression peaks. This coincidence suggests that NSUN7 helps to define the initiation sites for LC formation, which ultimately ensures their proper anchoring to the axoneme.

In both round and elongated spermatids, NSUN7 influences cytoplasmic proteins involved in dynein arm assembly, such as PIH1D3 and CCDC103 (round) and CCDC40 (elongated), as well as structural dynein components like DNALI1 and DNAH14. Distinguishing primary from secondary effects is challenging, but a plausible cascade is observed: the downregulation of PIH1D3 in round spermatids may cause the subsequent decrease in DNALI1 in mature spermatozoa, as *Pih1d3* knockout reduces DNALI1, specifically in sperm [29,42]. Notably, DNALI1 and CCDC103 knockouts produce a phenotype highly similar to *Nsun7^−/−^* mice, and DNALI1 has been shown to exert additional roles in transporting and assembling fibrous sheath proteins [28,39]. This parallel reinforces the idea that NSUN7 may participate in non-canonical pathways coordinating dynein function and fibrous sheath assembly.

Consistent with this, we also found that NSUN7 influences the protein abundance of intraflagellar transport (IFT) machinery components, such as IFT122, without altering their subcellular localization. IFT122 is a critical element of retrograde IFT-A trains that bridge IFT-A and IFT-B complexes [43]. IFT trains are organized at transition fibers, where they capture cytoplasmic and membrane cargo [44,45]. Interestingly, our data indicate that NSUN7 also stabilizes outer dense fiber protein 2 (ODF2), a core component of transition fibers, and several membrane-associated proteins. 

Another prominent feature of *Nsun7^−/−^* spermatozoa is the retention of residual cytoplasm [22]. Normally, cytoplasmic clearance relies on actin-driven tubulobulbar complexes [36,37], which are supported by a plectin scaffold that stabilizes endocytic sites [37,46]. Here, we show abnormal plectin accumulation in the residual cytoplasm of *Nsun7^−/−^* spermatids, suggesting impaired cytoskeletal remodeling. Interestingly, the localization of spectrin, which is also a component of the network surrounding tubulobulbar complexes [37], is not altered. This suggests that the observed morphological changes may be a direct outcome of a disrupted plectin level.

The changes in protein abundance between *Nsun7^−/−^* and wild-type spermatids are not static but develop progressively. The gradual depletion of fibrous sheath components (AKAP3, AKAP4, and ROPN1L) in *Nsun7^−/−^* compared to wild type illustrates this cumulative effect. Notably, these changes during spermatid development culminate in a proteome of mature spermatozoa that shows few differences from wild-type, suggesting that NSUN7 exerts its influence predominantly at earlier stages.

This progressive destabilization occurs without changes in mRNA levels for key effectors like AKAP4, CCDC103, and PIH1D3. A notable example is GSTM5 (Glutathione S-Transferase Mu 5); while NSUN7 binds Gstm5 mRNA without affecting its abundance [22], the GSTM5 protein level is reduced in *Nsun7^−/−^* sperm. Given evidence that NSUN7 lacks RNA methylation targets in spermatogenic cells [22,24,47], our data support a model where NSUN7 acts as a post-transcriptional regulator, likely stabilizing specific protein networks or enhancing their translation.

Thus, we propose that NSUN7 may act as a multifunctional structural scaffold during sperm development. Early on, it coordinates dynein assembly and IFT positioning, securing proper initiation and localization of LC. In elongating spermatids, it stabilizes fibrous sheath proteins (AKAP3, AKAP4, ROPN1L, DNALI1) and regulates cytoskeletal remodeling through plectin, enabling cytoplasmic clearance. Loss of NSUN7 destabilizes these networks, resulting in LC mislocalization, impaired flagellar function, and cytoplasmic retention. Importantly, these phenotypes are likely to stem from the absence of NSUN7’s non-enzymatic scaffold function rather than loss of methyltransferase activity [24].

Identified molecular networks have a strong association with human infertility. Mutations in several identified effector-proteins (DNALI1, CCDC103) were also shown to disrupt the positioning of LC [28,39]. Moreover, mutations in *NSUN7* cause significant abnormalities in the morphology of human sperm [19], which may be partially explained by disruption in plectin-associated processes.

Thus, NSUN7 emerges as a key regulator of sperm differentiation and cytoskeletal remodeling. Its structural role highlights a functional diversity within the NSUN family and suggests new targets for diagnosing and treatment of male infertility.

## 4. Materials and Methods

### 4.1. Mice Housing, Breeding, and Generation of Nsun7^−/−^

The experiments were conducted in strict accordance with the relevant national and international guidelines for the Care and Use of Laboratory Animals [48]. The animal study was carried out following the ARRIVE guidelines [49]. The work with animals was approved by the local bioethics committee “Institute of Mitoengineering MSU” LLC, protocol #79 dated 28 April 2015. The animals were housed in individually ventilated cages (IVC system, TECNIPLAST S.p.A., Buguggiate, Italy) with free access to food and water purified by reverse osmosis. The environment was free of specific pathogens and provided with light. The animals were housed in a 12/12 light cycle (light on at 09:00), in rooms with an air exchange rate exceeding 15 r/h, at 20–24 °C, and 30–70% humidity. Wood chips with minimal dust formation were utilized as bedding material. Shelters and building materials for nests were constructed from natural materials to enhance the environmental enrichment. All materials provided to the animals were sterilized by autoclaving.

The mouse line with the inactivation of the *Nsun7* gene was obtained previously [22]. Briefly, the inactivation of the *Nsun7* gene was performed by the CRISPR/Cas9 system and sgRNA with the guiding sequence ACACCGAGGCTGGAACAGCG, which was obtained by T7 transcription in vitro (MEGAscript™ T7 Transcription Kit, Thermofisher, Waltham, MA, USA). The obtained sgRNA was mixed with GeneArt™ CRISPR Nuclease mRNA (Thermofisher: A29378) and diluted in the filtered microinjection buffer (10 mM Tris, 0.1 mM EDTA, pH 8) to final concentrations of 25 ng/mL sgRNA and 50 ng/mL Cas9 mRNA.

Mice inbred strains C57Bl/6J and CBA (Federal Research Center Institute of Cytology and Genetics, Siberian Branch, Russian Academy of Sciences (ICG SB RAS) (Novosibirsk, Russia), SPF status, were mated to obtain F1 hybrids C57Bl/6J × CBA. Zygotes were obtained by mating the hybrid superovulated female mice with males using a standard procedure. Zygotes were microinjected into the pronucleus and transferred into the oviducts of pseudo-pregnant, commonly used outbred CD1 female mice. The CD1 mouse line was created from the ICR line in 1957 [50]. For further experiments, we selected the mutant mouse line with the insertion of 2 nt leading to a frameshift as the *Nsun7* knockout. To avoid any potential influence of unlikely secondary mutations, descendants of the founder were backcrossed to the inbred strain C57BL/6J. The wild-type and homozygous strains were obtained by crossing heterozygous mice *Nsun7^+/−^*. Wild-type mice were used to establish a reference control group.

The animals’ DNA samples from the tiny pieces of the distal tail (taking the tail biopsy was carried out in accordance with the FELASA July 2013 decision on genotyping of transgenic rodents) were extracted with QuickExtract™ DNA Extraction Solution (Lucigen: QE0905T, Middleton, WI, USA) according to the instructions of the manufacturer and analyzed by PCR and Sanger sequencing of the amplicons. Founder pups, their descendants, and wild-type and homozygous mice were genotyped by genomic DNA amplification with the primers TCAGATTTTCCATATTTAACACGAGTG and ATTTAGAAATCAAAACAAGTACCTTCG (knockouts for *Nsun7*), followed by Sanger sequencing (Center of Collective Use “Genome” at Engelhard Institute of Molecular Biology, Moscow, Russia). Experiments were performed on 2–6-month-old male mice. 

### 4.2. Sorting of Spermatogenic Cells

One biological replicate consisted of testes obtained from 3 mice from both the wild-type and *Nsun7^−/−^* mouse lines. Cell sorting was performed according to the previously published procedure [25]. Briefly, testes were decapsulated and incubated with collagenase type IV (2 mg/mL, Gibco, New York, NY, USA) at 37C for 20 min. After that seminiferous tubules were applied to the 5% Percoll solution and incubated on ice for 15 min. Seminiferous tubules, which had been drowned, were homogenized with Dounce homogenizer type A. The obtained cell suspension was twice filtered through a 40 μm cell strainer (Greiner Bio-One, Kremsmünster, Austria). After that cells were centrifuged (5 min, 800× *g*), dissolved in 3 mL of PBS, and fixed on ice for 15 min with 3 volumes of ethanol 100% supplied with 0.8 mM EDTA. Then cells were stained with SYTO16 (Invitrogen, Waltham, MA, USA) on ice for 30 min.

Cell sorting was performed on a 4-Laser (405 nm—violet, 488 nm—blue, 561 nm—yellow-green, 633 nm—red) 20-parameter BD FACSAria III, and the BD FACSDiva 6.1.3 software was used to visualize and analyze the data. Laser delay was set to 0 for the blue, −77.39 for the red, 37.33 for the violet, and −39.85 for the yellow-green lasers. Area scaling was set to 1.14 for the blue, 1.0 for the red, 0.75 for the violet, and 0.96 for the yellow-green lasers. Window extension was set to 2.00 µs, and the FSC Area scaling to 1.00. A sample filter line of 50 µm was placed at the end of the sample line to avoid clumping.

Cells on 1–9 (round spermatids) and 10–16 (elongated spermatids) steps of spermatogenesis were collected in falcons covered with FBS. 

The mature spermatozoa were collected from the epididymis of wild-type (*n* = 3) and *Nsun7^−/−^* (*n* = 3) mice.

### 4.3. Proteome Analysis

Spermatozoa, round spermatids, and elongated spermatids collected from wild-type (*n* = 3) and *Nsun7^−/−^* (*n* = 3) mice lice were lysed in lysis buffer (5% SDS, 0.1% SDC, 100 mM TEAB) with protease inhibitor cocktail (Thermofisher) and ultrasonicated (3 cycles for 10 sec of ultrasonication on Ultrasonic professor, Cole-Parmer, Vernon Hills, IL, USA). The protein concentration was measured with DC™ Protein Assay Kit II (Biorad, Hercules, CA, USA). Briefly, 100 μg of proteins were taken for further analysis. Reduction, alkylation, and trypsin (Promega, Madison, WI, USA) digestion were conducted under manufacture recommendations of the S-Trap™ micro-MS sample prep kit (Protifi Innovations omics solutions, Fairport, NY, USA). The obtained peptides were collected under centrifugation.

The sample peptides, in a volume of 2 µL, were loaded onto the Acclaim µ-Precolumn (0.5 mm × 3 mm, 5 µm particle size, Thermofisher) at a flow rate of 15 µL/min for 4 min in an isocratic mode of Mobile Phase C (2% acetonitrile, 0.1% formic acid). Then the peptides were separated with high-performance liquid chromatography (HPLC, Ultimate 3000 Nano LC System, Thermofisher) in a 25 cm long C18 column (Peaky C18 column, inner diameter of 100 μm, Molecta, Moscow, Russia). The peptides were eluted with a gradient of buffer B (80% acetonitrile, 0.1% formic acid) at a flow rate of 0.4 μL/min. The total run time was 90 min, which included an initial 4 min of column equilibration to buffer A (0.1% formic acid), then a gradient from 5 to 35% of buffer B over 65 min, then 6 min to reach 99% of buffer B, flushing for 10 min with 99% of buffer B, and 5 min re-equilibration to buffer A.

Mass spectrometry (MS) analysis is an analytical technique for identifying different compounds by measuring their mass-to-charge ratio. MS analysis was performed at least in triplicate with a Q Exactive HF-X mass spectrometer (Q Exactive HF-X Hybrid Quadrupole-OrbitrapTM Mass spectrometer, Thermofisher). The temperature of the capillary was 250 °C, and the voltage at the emitter was 2.1 kV. Mass spectra were acquired at a resolution of 60,000 (MS) in a range of 400–1500 m/z. Tandem mass spectra of fragments were acquired at a resolution of 15,000 (MS/MS) in the range from 140 m/z to the m/z value determined by a charge state of the precursor. The maximum integration time was 50 ms and 30 ms for precursor and fragment ions, respectively. The AGC target for precursor and fragment ions was set to 1 × 106 and 1 × 105, respectively. An isolation intensity threshold of 100,000 counts was determined for precursor selection, and up to the top 20 precursors were chosen for fragmentation with high-energy collisional dissociation (HCD) at 29 NCE. Precursors with a charge state of +1 and more than +5 were rejected, and all measured precursors were dynamically excluded from triggering of a subsequent MS/MS for 50 s.

The obtained raw data files were processed with the MaxQuant software (version 2.1.0.0) [51] using the internal search engine Andromeda [52] and searched against the UniProtKB database restricted to Mus musculus (51,444 entries). For the quantification, the same parameters were used as in the previous study [53]. Briefly, for the peptide, the following parameters were set: trypsin digestion only with a maximum of two missed cleavages, minimum length—six amino acids. N-terminal acetylation and methionine oxidations were set as variable modifications, and cysteine carbamidomethylation as fixed. The fragment mass tolerance was 0.05 Da, and the precursor mass tolerance was 20 ppm. The Match between run function was enabled. Label-free quantification (LFQ) was estimated with the MaxLFQ algorithm [54], and a minimum ratio count of 1 was set. The FDR for protein identification was set to 0.01, and at least two unique peptides were required.

Bioinformatic analysis of the identified and quantified data was performed with the DEP package (version 1.23.0) [55] on R (version 4.1.2). Statistical significance was determined using the protein-wise linear models and empirical Bayes statistics [56]. For filtration of differentially expressed proteins following thresholds were set: an adjusted *p* value less than 0.05 and a fold change (FC) greater than 1.5.

To analyze trends in protein amount changes, we selected proteins for which FC was gradually decreasing or increasing throughout the three investigated stages, and the ratio between FC in round spermatids and spermatozoa was above 2.

Bioinformatic analysis of GO term enrichment was performed with ShinyGO V0.81 [57].

### 4.4. Targeted Mass-Spectrometry Analysis of NSUN7

Targeted mass-spectrometry analysis of NSUN7 was performed according to the previously described procedure [22]. Solid-phase synthesis of stable isotope-labeled standard (SIS) TVSQAGTSSQVR was carried out on an Overture automated peptide synthesizer (Protein Technologies, Manchester, UK). In the synthesis of SIS peptides, isotope-labeled amino acids Fmoc-Arg-OH-13C6,15N2 (Cambridge Isotope Laboratories, Tewksbury, MA, USA) were incorporated in peptide sequences instead of the native counterparts. Experimental samples were spiked with SIS at a concentration of 10 fmole per μg of total peptides.

Targeted mass spectrometric analysis in the selected reaction monitoring (SRM) mode was carried out according to the following protocol. Each experimental sample was analyzed in three technical replicates. Chromatographic separation was carried out using an Agilent 1200 series system (Agilent Technologies, Santa Clara, CA, USA) coupled to a TSQ Quantiva triple quadrupole mass analyzer (Thermofisher, Waltham, MA, USA). A 3.5 μL sample containing 7 μg of native peptides and SIS standards was separated using a ZORBAX SB-C18 analytical column (150 × 0.5 mm, 5 μm particle diameter, Agilent Technologies) in an acetonitrile gradient at a flow rate of 20 μL/min. The column was initially equilibrated with 5% (*v*/*v*) solution B (80% (*v*/*v*) acetonitrile in 0.1% (*v*/*v*) formic acid) and 95% (*v*/*v*) solution A (0.1% (*v*/*v*) formic acid) for 5 min, then the concentration of solution B was linearly increased to 60% (*v*/*v*) over 30 min, after which the concentration of solution B was increased to 99% (*v*/*v*) over 1 min and the column was washed with 99% (*v*/*v*) solution B for 5 min, then the concentration was returned to the initial conditions over 1 min, in which the column was equilibrated for 9 min. Mass spectrometric analysis was performed in the dynamic selected reaction monitoring (dSRM) mode using the following MS detector settings: capillary voltage was 4000 V, drying gas flow rate (nitrogen) was 7 L/min, axillary gas flow rate (nitrogen) was 5 L/min, capillary temperature was 350 °C, isolation window for the first and third quadrupoles was 0.7 Da, scan cycle time was 1.2 s, gas pressure (argon) in the collision cell was 1.5 mTorr. SRM transitions are listed in Table “SRM Table”. All transitions from which the signal was recorded were used for quantification in Skyline software (version 4.1.0).

Mass-spectrometry measurements were performed using the equipment of the “Human Proteome” Core Facilities of the Institute of Biomedical Chemistry (Moscow, Russia).

### 4.5. Immunohistochemistry

Paraffin sections were washed with xylene three times, then dehydrated in two changes of 100% pure ethanol, followed by overnight rehydration in TBS (10 mM Tris-HCl, pH 7.5, 150 mM NaCl). For permeabilization, tissue sections were incubated in a citric buffer (10 mM citric acid, 0.05% Tween-20) for 12 min at 96 °C in a stirred water bath WB-4MS (Biosan, Novosibirsk, Russia). Blocking was conducted in TBS supplied with 2% bovine serum albumin (BSA, Proliant Biologicals, Ankeny, IA, USA). The following primary antibodies were used in the corresponding dilutions: anti-α-tubulin Alexa 488 (1:200, ab18251, Abcam, Cambridge, UK), anti-acetylated α-tubulin Alexa 488 (1:100, K0821, Santa Cruz Biotechnology, Dallas, TX, USA), anti-PLEC (1:100, MA5-32102, Thermofisher), anti-β-Spectrin II (1:50, 612562, BD Transduction Laboratories, Lexington, KY, USA), and anti-PIH1D3 (1:200, 25309-1-AP, Proteintech, Rosemont, IL, USA). For detection, the following secondary antibodies were used: Cy5 Goat anti-Rabbit (1:500, A10523, Thermofisher), anti-mouse Cy5 (1:500, A10524, Thermofisher), anti-rabbit FITC (1:200, ab7050, Abcam). Tissue sections were covered with Mowiol supplied with DAPI (Thermofisher).

### 4.6. Immunocytochemistry

Spermatogenic cells were obtained from testes, which were decapsulated and incubated with collagenase type IV (2 mg/mL, Gibco) at 37C for 20 min. After that seminiferous tubules were applied to the 5% Percoll solution and homogenized with Dounce homogenizer type A. The obtained cell suspension was twice filtered through a 40 μm cell strainer (Greiner Bio-One). After that cells were centrifuged (5 min, 800× *g*), dissolved in 1 mL of PBS. Cell suspension was applied on poly-L-lysine slides and fixed with ice-cold methanol for 20 min at room temperature. Blocking was conducted in TBS supplied with 2% bovine serum albumin (BSA, Proliant Biologicals). The following primary antibodies were used in the corresponding dilutions: anti-acetylated α-tubulin Alexa 488 (1:100, K0821, Santa Cruz Biotechnology), anti-PLEC (1:100, MA5-32102, Thermofisher), and anti-IFT122 (1:100, ab111838, Abcam). For detection, the following secondary antibodies were used: Cy5 Goat anti-Rabbit (1:500, A10523, Thermofisher). Samples were covered with Mowiol supplied with DAPI (Thermofisher).

### 4.7. Immunoblotting

Immunoblotting of tissue lysates was performed as previously described [22]. For immunoblot analysis, sample-sorted cell fractions were lysed in lysis buffer (5% SDS, 0.1% SDC, 100 mM TEAB) with protease inhibitor cocktail (Thermofisher). The transfer-ready PVDF membrane (Thermofisher) was blocked for 1 h in TBST (10 mM Tris-HCl, pH 7.5, 150 mM NaCl, 0.1% Tween-20) containing 5% bovine serum albumin (BSA, Proliant Biologicals). The following primary antibodies were used: anti-CCDC103 (1:1000, PA5-113107, Thermofisher), anti-PLEC (1:1000, MA5-32102, Thermofisher), anti-IFT122 (1:1000, ab111838, Abcam), anti-AKAP4 (1:1000, PA5-109377, Thermofisher).

The secondary HRP-conjugated anti-rabbit (1706515, Biorad) antibodies were used at a 1:5000 dilution. anti-α-Tubulin (1:3000, ab18251, Abcam) was used as a loading control.

## Data Availability

The mass spectrometry proteomics data have been deposited in the ProteomeXchange Consortium via the PRIDE partner repository with the dataset PXD067398 (round spermatids), PXD067394 (elongated spermatids), PXD067410 (spermatozoa). SRM data have been deposited in the ProteomeXchange Consortium via the PRIDE partner repository with the dataset PXD069148.

## Supplementary Materials

The following supporting information can be downloaded at: https://www.mdpi.com/article/10.3390/ijms27010257/s1.
