# Supplementary material for: NSUN7 Regulates Sperm Flagella Formation at All Stages of Spermiogenesis
Source: Int J Mol Sci. 2025 Dec 25;27(1):257. doi: 10.3390/ijms27010257 (PMC12785897; doi:10.3390/ijms27010257)
Supplement: Supplementary file 1 [file ijms-27-00257-s001.zip › ijms-4001769-supplementary.pdf]

Supplementary figures

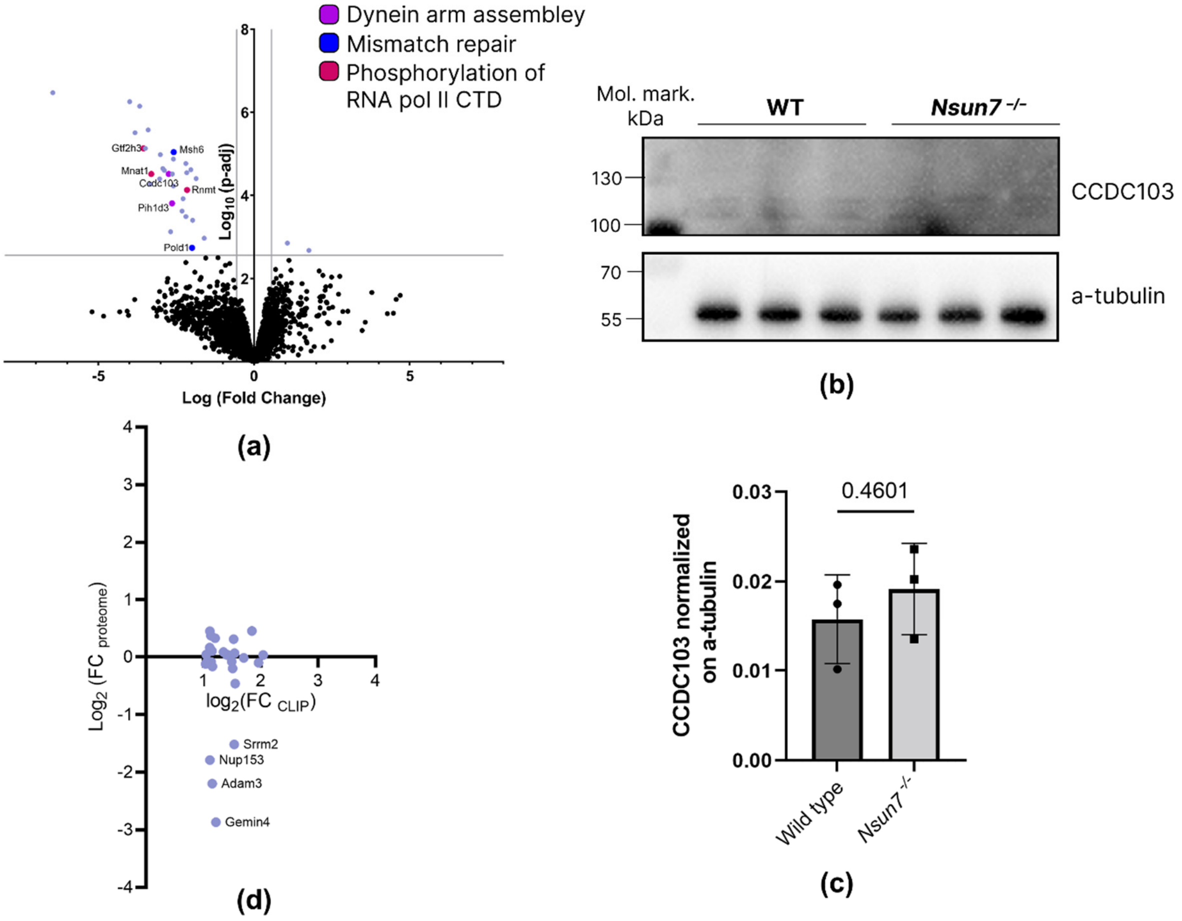

Figure S1. (a) Volcano plot showing DEPs of round spermatids in the wild type (n=3) and *Nsun7*$^{-/-}$ (n=3) mice colored by GO term annotation. (b) Comparative immunoblotting of elongated spermatid lysates of wild type (WT, left 3 lanes) and *Nsun7*$^{-/-}$ (right 3 lanes) mice. Antibodies against CCDC103 and control antibodies against α-tubulin were used as indicated right to the panels. (c) Bar graph showing α-tubulin normalized levels IFT122 in round spermatids of wild type and *Nsun7*$^{-/-}$ mice according to immunoblotting. Means ± standard deviation are shown (n=3). (d) Correlation between efficiency of RNA cross-linking to the NSUN7 as determined by CLIP-Seq [1] and fold change (FC) in proteome of round spermatids of wild type and *Nsun7* $^{-/-}$ mice.

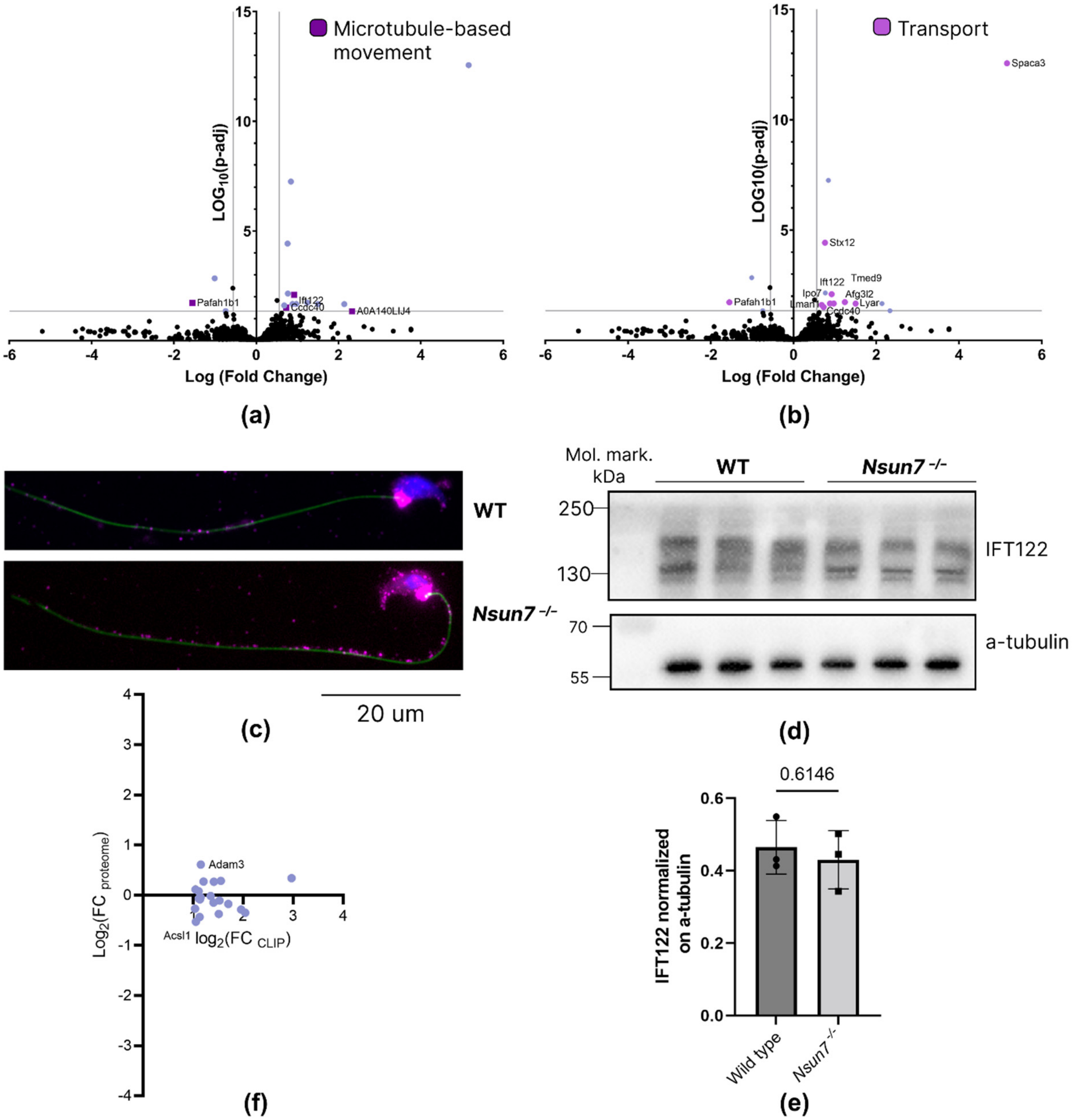

Figure S2. (a-b) Volcano plot showing DEPs associated with microtubule-based movement (a) and with transport (b) of elongated spermatids in the wild type (n=3) and *Nsun7*$^{-/-}$ (n=3) mice. (c) Immunostaining of elongated spermatids of wild type and *Nsun7*$^{-/-}$ mice with anti-IFT122 antibodies. (d) Comparative immunoblotting of round spermatid lysates of wild type (left 3 lanes) and *Nsun7*$^{-/-}$ (right 3 lanes) mice. Antibodies against IFT122 and control antibodies against α-tubulin were used as indicated right to the panels. (e) Bar graph showing α-tubulin normalized levels IFT122 in round spermatids of wild type and *Nsun7*$^{-/-}$ mice according to

immunoblotting. Means ± standard deviation are shown (n=3). (f) Correlation between efficiency of RNA cross-linking to the NSUN7 as determined by CLIP-Seq [1] and fold change (FC) in proteome of elondated spermatids of wild type and *Nsun7* $^{-/-}$ mice.

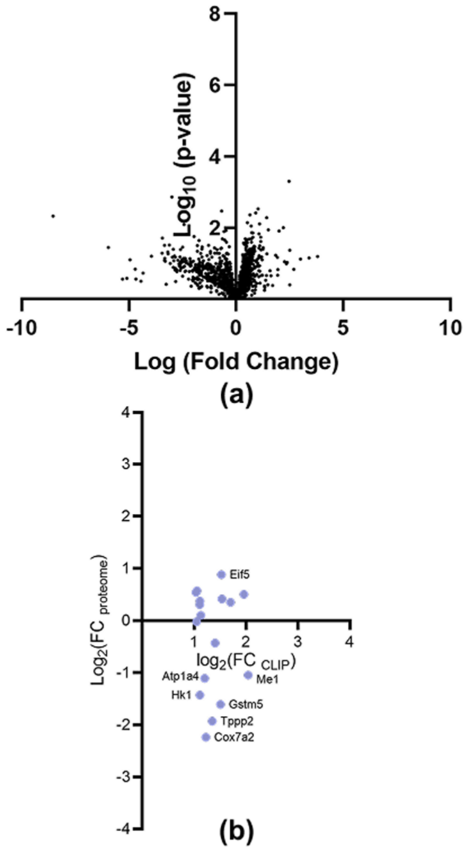

Figure S3. (a) Volcano plot showing proteins of mature spermatozoa in the wild type (n=3) and *Nsun7* $^{-/-}$ (n=3) mice colored by GO term annotation. (b) Correlation between efficiency of RNA cross-linking to the NSUN7 as determined by CLIP-Seq [1] and fold change (FC) in proteome of spermatozoa of wild type and *Nsun7* $^{-/-}$ mice.

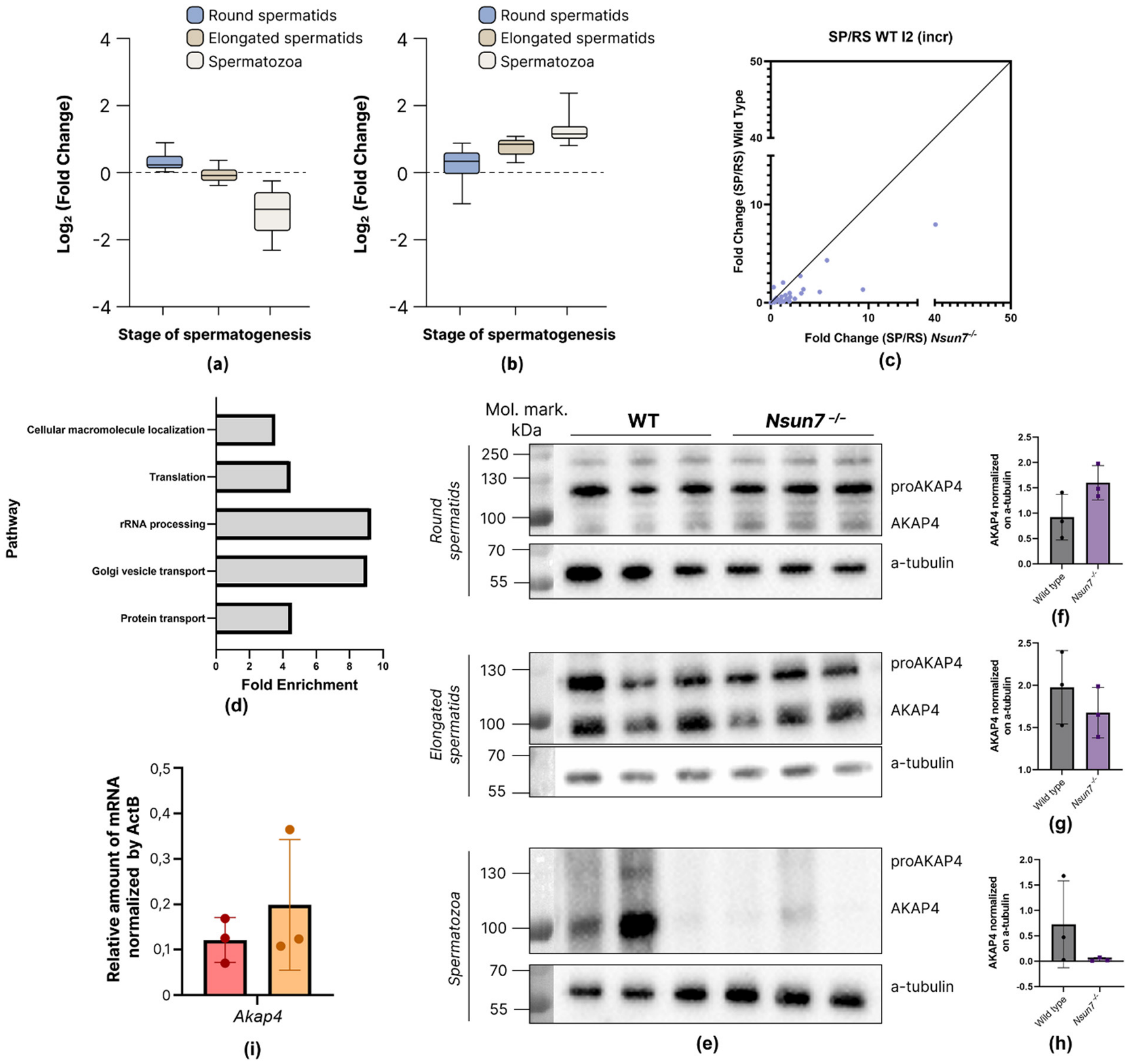

Figure S4. (a-b) Decreased and increased trends in Fold Changes of proteins during spermatogenesis. (c) Scatter plot of Fold Changes in protein amount between round spermatids and spermatozoa in wild type and *Nsun7*$^{-/-}$ mice. (d) GO term enrichment analysis of the proteins with decreasing trends in FC changes. (e) Comparative immunoblotting of spermatozoa, round and elongated spermatid lysates of wild type (left 3 lanes) and *Nsun7*$^{-/-}$ (right 3 lanes) mice. Antibodies against AKAP4 and control antibodies against α-tubulin were used as indicated right to the panels. (f-h) Bars showing α-tubulin normalized levels AKAP4 in spermatozoa (h), round (f) and elongated (g) spermatids of wild type and *Nsun7*$^{-/-}$ mice according to immunoblotting (c).

Means ± standard deviation are shown (n=3). (i) Comparison of the mRNA levels of Akap4 in wild type and *Nsun7*$^{-/-}$ mice.

Original immunoblotting images

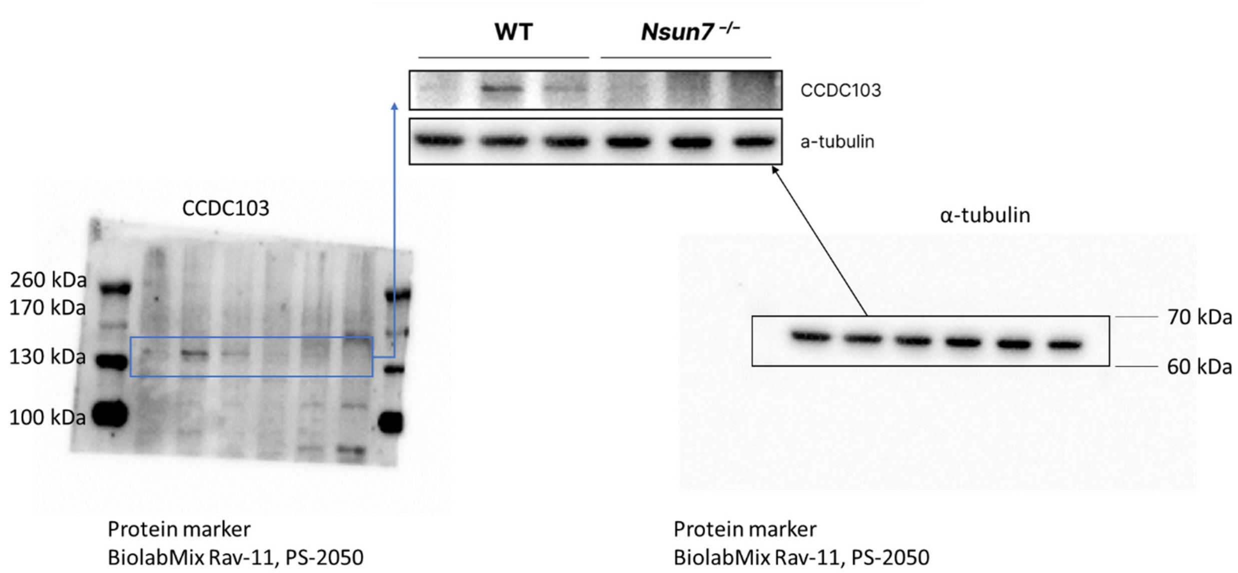

Figure S5. Original uncut images corresponding to the Figure 4A: Comparative immunoblotting of round spermatid lysates of wild type (left 3 lanes) and *Nsun7*$^{-/-}$ (right 3 lanes) mice. Antibodies against CCDC103 and control antibodies against α-tubulin were used as indicated right to the panels.

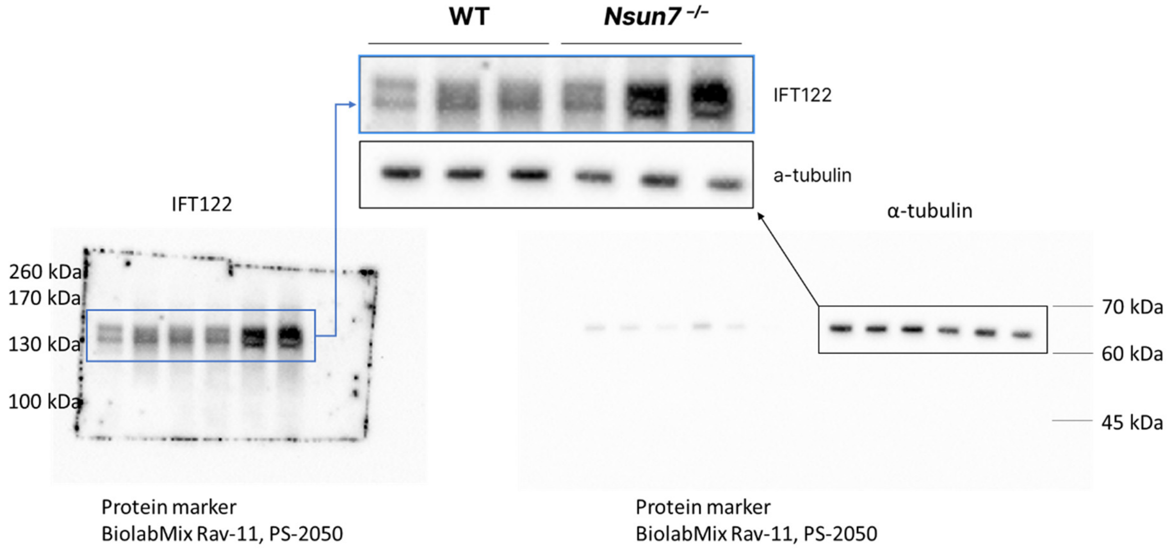

Figure S6. Original uncut images corresponding to the Figure 5A: Comparative immunoblotting of elongated spermatid lysates of wild type (left 3 lanes) and Nsun7-/- (right 3

lanes) mice. Antibodies against IFT122 and control antibodies against α-tubulin were used as indicated right to the panels.

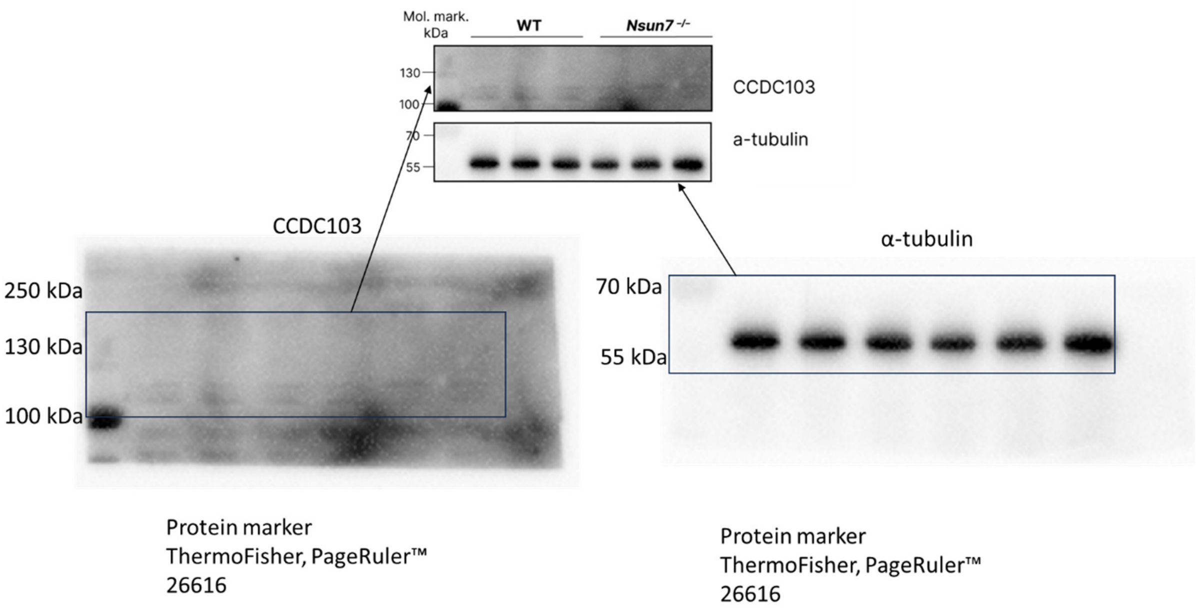

Figure S7. Original uncut images corresponding to the Figure S1B: Comparative immunoblotting of elongated spermatid lysates of wild type (left 3 lanes) and Nsun7-/- (right 3 lanes) mice. Antibodies against CCDC103 and control antibodies against α-tubulin were used as indicated right to the panels.

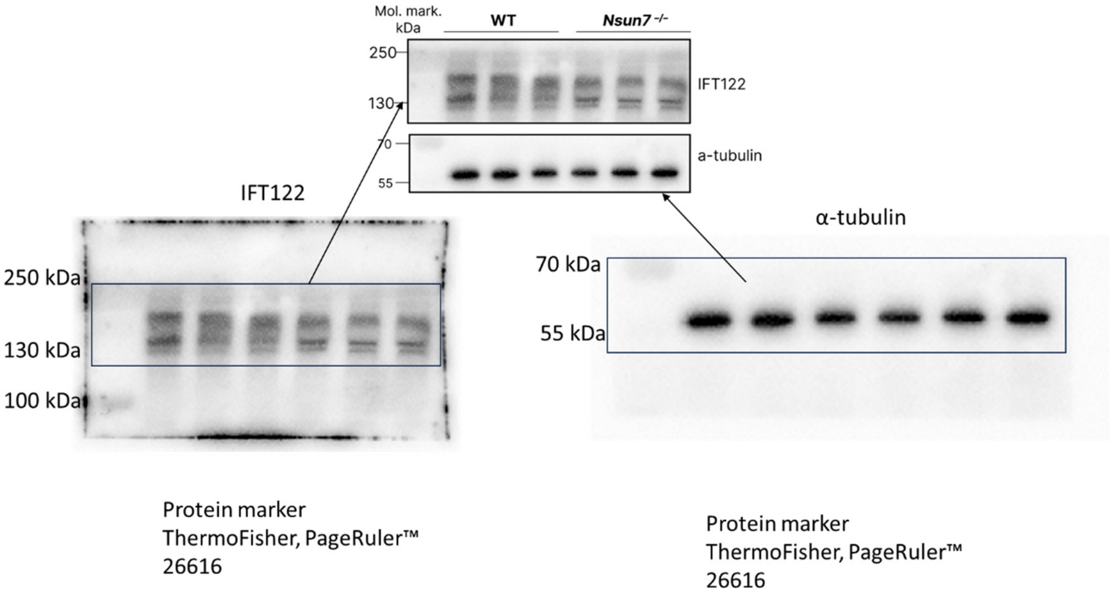

Figure S8. Original uncut images corresponding to the Figure S2D: Comparative immunoblotting of round spermatid lysates of wild type (left 3 lanes) and Nsun7-/- (right 3 lanes) mice. Antibodies against IFT122 and control antibodies against α-tubulin were used as indicated right to the panels.

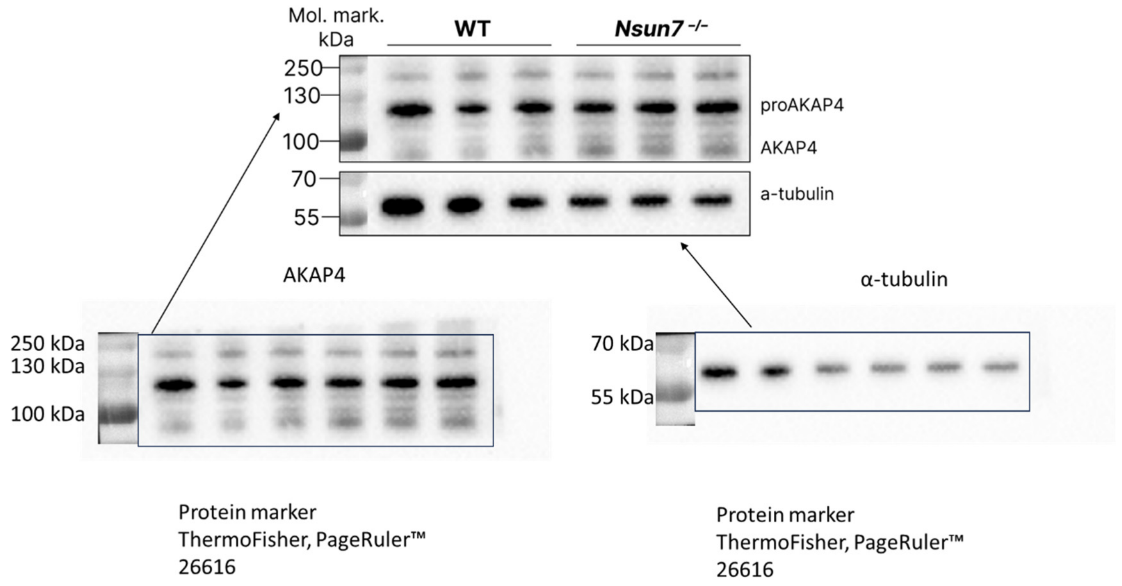

Figure S9. Original uncut images corresponding to the Figure S4E: Comparative immunoblotting of round spermatid lysates of wild type (left 3 lanes) and Nsun7-/- (right 3 lanes) mice. Antibodies against AKAP4 and control antibodies against α-tubulin were used as indicated right to the panels.

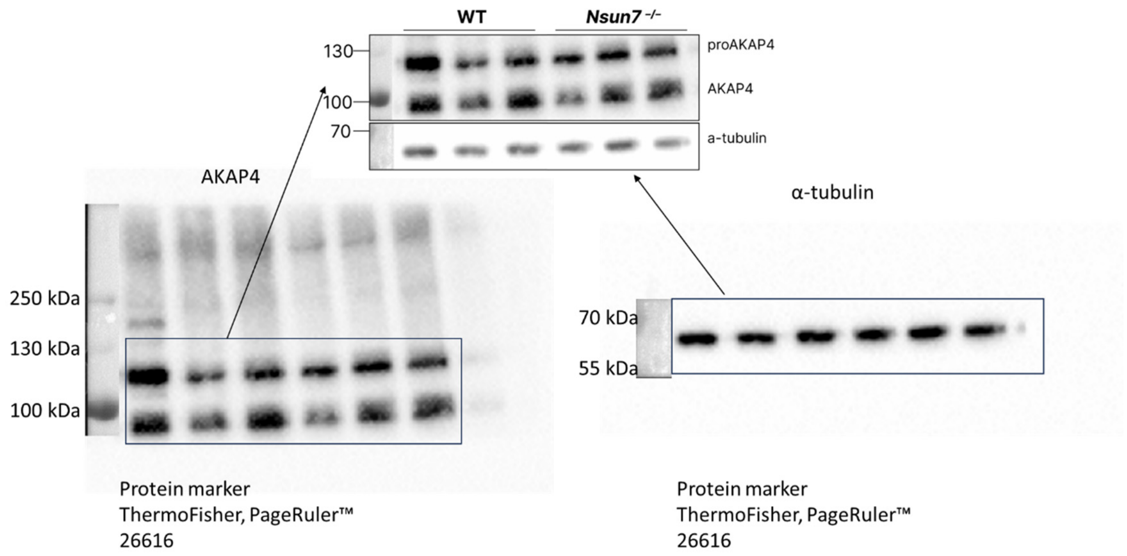

Figure S10. Original uncut images corresponding to the Figure S4E: Comparative immunoblotting of elongated spermatid lysates of wild type (left 3 lanes) and Nsun7-/- (right 3 lanes) mice. Antibodies against AKAP4 and control antibodies against α-tubulin were used as indicated right to the panels.

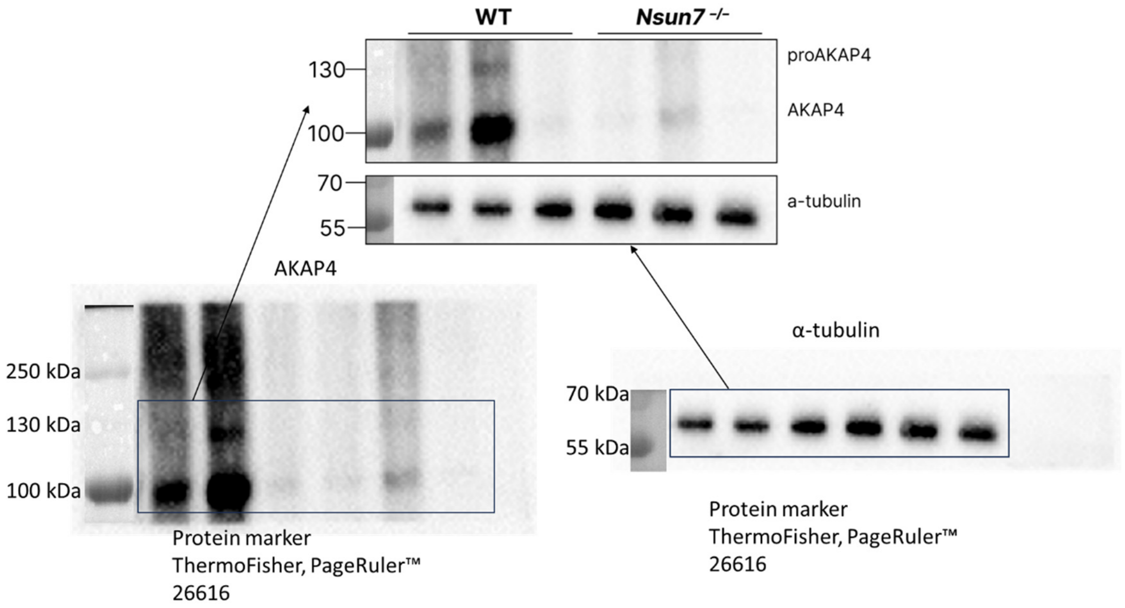

Figure S11. Original uncut images corresponding to the Figure S4E: Comparative immunoblotting of spermatozoa lysates of wild type (left 3 lanes) and Nsun7-/- (right 3 lanes) mice. Antibodies against AKAP4 and control antibodies against α-tubulin were used as indicated right to the panels.

References:

1. Guseva, E.A.; Averina, O.A.; Isaev, S.V.; Pletnev, P.I.; Bragina, E.E.; Permyakov, O.A.; Buev, V.S.; Priymak, A.V.; Emelianova, M.A.; Pshanichnaya, L.; et al. Positioning of Sperm Tail Longitudinal Columns Depends on NSUN7, an RNA-Binding Protein Destabilizing Elongated Spermatid Transcripts. *RNA N. Y. N* **2025**, *31*, 709–723, doi:10.1261/rna.080320.124.
